# Contrasting PSII Photochemistry and Energy Partitioning Between Spikes and Leaves During Grain Anthocyanin Accumulation in Hulless Barley on the Tibetan Plateau

**DOI:** 10.3390/plants15101489

**Published:** 2026-05-13

**Authors:** Zhongmengyi Qin, Xiaoxia Yang, Shuaihao Chen, Hongkang Zhou, Yetao Wang, Yutong Zheng, Liping Niu, Dawa Dondup, Xin Hou

**Affiliations:** 1 School of Ecology and Environment, Tibet University, Lhasa 850000, China; qinzmy4205@163.com (Z.Q.); lipingniu@whu.edu.cn (L.N.); 2 College of Life Sciences, Wuhan University, Wuhan 430072, China; yangxiaoxia7@fafu.edu.cn (X.Y.); csh102050@126.com (S.C.); 2023202040160@whu.edu.cn (H.Z.); 2016202040073@whu.edu.cn (Y.W.); ytzheng@whu.edu.cn (Y.Z.); 3 College of Agriculture, Fujian Agriculture and Forestry University, Fuzhou 350002, China; 4 State Key Laboratory of Hulless Barley and Yak Germplasm Resources and Genetic Improvement, Lhasa 850002, China; dwdunzhu@126.com

**Keywords:** grain color, spike, anthocyanin, photosynthetic adaptation, hulless barley

## Abstract

Hulless barley (*Hordeum vulgare* L. var. *nudum*) on the Qinghai–Tibet Plateau is consistently exposed to intense solar irradiance, yet whether and how reproductive spikes and flag leaves partition photoprotection remains unclear. Here, we compared a pigmented black landrace (Cai Peng Zi, CPZ) with a white cultivar (Zang Qing 3000, ZQ3000) across early, middle, and late spike coloration stages under field conditions. By integrating measurements of anthocyanin and chlorophyll contents, chlorophyll fluorescence parameters, and rapid light-response curves, we dissected organ-specific strategies in photochemistry and energy dissipation in spikes and flag leaves. The results showed that anthocyanin accumulation in CPZ spikes increased significantly during spike coloration, while chlorophyll a and the chlorophyll a/b ratio declined, indicating a shift from light harvesting to photoprotection in reproductive tissues. This pigment transition coincided with reduced PSII performance (declines in QY_max_, qP, and qL) but stable non-photochemical quenching (NPQ and qN), pointing to reduced photochemical capacity with relatively stable energy dissipation in the spike. In contrast, CPZ leaves maintained higher QY_max_ than ZQ3000 but exhibited a pronounced decline in NPQ and qN at late stages, reflecting CPZ’s attenuated regulated energy dissipation capacity. Rapid light-response analysis further supported differences between organs and cultivars. Under high PAR, ZQ3000 spikes exhibited steeper declines in Y(II) and stronger downregulation of ETR(II), whereas CPZ spikes showed more moderate decreases; in leaves, ZQ3000 maintained consistently lower Y(NO) and higher Y(NPQ), indicating greater reliance on regulated energy dissipation. Collectively, our results reveal how pigment-mediated screening in reproductive structures and dynamic regulation of energy dissipation in leaves are coordinated to optimize light-use efficiency in high-altitude environments, providing physiological insights for breeding resilient hulless barley varieties.

## 1. Introduction

Hulless barley (*Hordeum vulgare* L. var. *nudum*), locally known as Qingke in China, is the most important cereal crop on the Tibetan Plateau, where it has been cultivated for more than 3500 years [1,2]. It plays a dual role as a staple food for humans and feed for livestock, underpinning the plateau’s agroecosystem [3]. The species has evolved remarkable tolerance to the Plateau’s extreme environmental conditions, including intense solar radiation, low temperature, and drought [4,5,6,7]. Over the past decade, Qingke has attracted growing interest as a functional food owing to its health benefits [8,9].

Different colored hulless barley cultivars have been widely cultivated in Tibet [10,11]. Hulless barley exhibits remarkable color diversity in its grains, ranging from white to blue, purple, and black [12,13]. Black barley is widely cultivated in Tibet owing to its resistance to biotic and abiotic stresses [14]. In natural field conditions on the Tibetan Plateau, the spikes of black barley often undergo a distinct color transition, shifting from green to purple before harvest. This phenomenon implies an organ-level physiological adjustment involving pigment metabolism and photoprotection. Anthocyanins constitute one of the major groups of natural pigments, which are responsible for the colors of many fruits and flowers [15]. Among the diverse cultivars of Qingke, the black barley landrace exhibits a distinctive purple to black coloration in grains and spikes, a trait associated with the accumulation of anthocyanins and other flavonoid pigments [16,17]. Anthocyanins are water-soluble flavonoid polyphenol pigments present in many plant foods, including red grapes, red cabbage, anthocyanin-rich barley, rice, and maize [18,19,20,21,22]. Evidence from observational studies and randomized trials suggests potential health benefits of anthocyanin intake. Adding anthocyanin-rich black hulless barley to the daily diet can positively impact cardiovascular health by lowering triglycerides and increasing high density lipoprotein [23]. The distribution of black and purple phenotypes correlates with altitude and solar radiation intensity, suggesting an ecological link between pigmentation and adaptation to high-UV environments [6,24]. Genomic studies have revealed that several gene families in Qingke underwent adaptive expansion, conferring enhanced resilience to high ultraviolet (UV-B) radiation and hypoxic stress at high altitudes [4].

Anthocyanins not only determine visible coloration but also play critical photoprotective roles under high-light or UV-B exposure by attenuating excess light energy, scavenging reactive oxygen species, and stabilizing photosynthetic machinery [15,25]. This optical screening function is largely attributed to the strategic localization of these pigments within epidermal and subepidermal vacuoles, where they intercept incoming photons before they reach the underlying mesophyll [26,27]. Anthocyanins shield the photosynthetic apparatus from excess light photons that would otherwise be captured by antenna complex pigments. Meanwhile, red leaves absorb substantial amounts of light via non-photosynthetic pigments such as anthocyanins. Consequently, the photosynthetic tissues of red leaves receive fewer light quanta than those of green leaves [28,29]. These studies establish anthocyanin screening as a recognized photoprotective mechanism in vegetative tissue. Anthocyanin biosynthesis is known to be regulated by environmental cues such as light intensity, temperature, and oxidative stress, through transcriptional activation of MYB–bHLH–WD40 complexes controlling flavonoid pathway genes [24,30]. Concurrently, the degradation of chlorophyll and remodeling of pigment–protein complexes represent essential processes in maintaining the balance between light harvesting and photoprotection during developmental or stress-induced transitions [31,32]. In cereal flag leaves, this remodeling is not a mere byproduct of decay but a coordinated physiological process. Field observations in barley and wheat indicate that while components like cytochrome *f* and Rubisco decline post-anthesis, the light-harvesting complexes are retained longer or dismantled in distinct kinetic phases to mitigate photoinhibition [33,34,35]. These findings indicate that pigment turnover during reproductive development is not a uniform decline but a coordinated remodeling that affects photochemistry and photoprotection along distinct temporal trajectories.

While extensive research has focused on anthocyanin accumulation in leaves, fruits, and grains, the photosynthetic responses of reproductive organs, such as spikes or ears, remain much less understood, particularly under high-altitude field conditions [36,37,38]. In cereal crops like wheat and barley, spikes possess significant photosynthetic capacity, contributing up to 20–40% of total grain carbon assimilation [39]. The specialized structures of awns and glumes facilitate light capture, heat dissipation, and water-use efficiency, enabling the spike to maintain photosynthetic activity even under drought or high-radiation stress [40]. Moreover, the delayed senescence of spike tissues supports sustained photosynthesis during late grain filling, serving as a physiological “backup system” for carbon supply under environmental constraints [41,42]. However, the photosynthetic dynamics of the spike during its color transition, particularly in comparison with leaves, remain poorly understood in black barley. Specifically, it is unclear how pigment changes affect photosystem II (PSII) activity, photochemical efficiency, and non-photochemical quenching mechanisms across different organs. Furthermore, the potential coordination between leaf and spike photosynthesis during the transition period has not been systematically examined. Addressing these questions is essential for understanding how highland crops optimize light utilization and energy balance in environments characterized by intense radiation and fluctuating temperature.

Here, we investigated the organ-specific photosynthetic responses of black hulless barley landrace (CPZ) during its natural color transition process under field conditions on the Qinghai–Tibet Plateau. Using a combination of pigment quantification, chlorophyll fluorescence imaging, and rapid light response analysis, we compared the dynamic changes in photochemical parameters between leaves and spikes across different color transition stages. Through a comparative study with a non-pigmented white hulless barley cultivar (ZQ3000), we aimed to elucidate the relationship between anthocyanin accumulation, chlorophyll degradation, and photosynthetic efficiency, assess the organ-specific photochemical strategies in leaves and spikes under high-light stress, and uncover the coordinated regulatory mechanisms underlying organ-level adaptation during color transition. Because ZQ3000 produces no visible spike pigmentation that could be used as an analogous staging anchor, the inter-genotype contrast is time-matched rather than phenology-matched. We therefore treat genotype-level differences as descriptive of two cultivars co-existing under identical field conditions. This study provides new insights into the photosynthetic plasticity and photoprotective strategies of hulless barley. Understanding how pigment-mediated modulation of photosynthesis contributes to stress tolerance at the organ level will deepen our comprehension of plant adaptation to intense solar radiation, and guide breeding for improved light-use efficiency in crops exposed to abiotic stress.

## 2. Results

### 2.1. Variations in Pigment Content Associated with Spike Coloration in Hulless Barley

**Figure 1:**
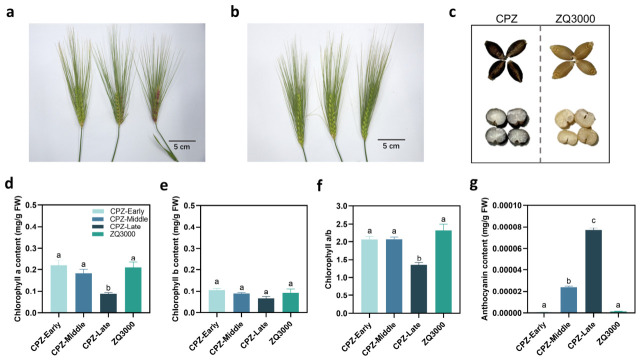
Phenotypic characteristics and pigment content changes during the spike coloration process in black hulless barley CPZ and white hulless barley ZQ3000. (**a**) Phenotypic images of black hulless barley CPZ at the early, middle, and late stages of spike coloration. (**b**) Phenotypic images of white hulless barley ZQ3000 during the corresponding stages of spike coloration. Scale bar = 5 cm. (**c**) External morphology and transverse sections of mature grains from black hulless barley (CPZ) and white hulless barley (ZQ3000). (**d**) Changes in chlorophyll a content at different stages of coloration in CPZ and ZQ3000 spikes. (**e**) Changes in chlorophyll b content at different stages of coloration in CPZ and ZQ3000 spikes. (**f**) Comparison of the chlorophyll a/b ratio between CPZ and ZQ3000 during spike coloration. (**g**) Anthocyanin content in spikes. Data are shown as mean ± SE (*n* = 3). One-way ANOVA with Tukey’s test or Kruskal–Wallis with Dunn’s test was used depending on data distribution. Different lowercase letters indicate significant differences between groups at *p* < 0.05. The same letter indicates no significant difference (*p* > 0.05).

We first characterized the phenotypic and physiological changes during spike coloration in the black hulless barley landrace CPZ and the white cultivar ZQ3000 (Figure 1). Specifically, the progressive color changes in CPZ spikes from green to purplish across the early, middle, and late stages are shown in representative images (Figure 1a). In contrast, ZQ3000 spikes consistently remained green, with no visible coloration observed (Figure 1b). Images taken under standardized conditions clearly revealed differences in external grain morphology and transverse section structures between CPZ and ZQ3000 (Figure 1c). Compared to ZQ3000, which showed a pale yellow to nearly colorless tissue structure throughout the kernel, CPZ grains exhibited strong pigmentation in the aleurone layer, with slight coloration also observed in the endosperm.

To investigate pigment dynamics during spike coloration, we analyzed anthocyanin accumulation and chlorophyll degradation in CPZ and ZQ3000. Anthocyanin content increased significantly in CPZ spikes at the middle and late stages compared to the early stage and to ZQ3000 (*p* < 0.05), indicating that enhanced anthocyanin biosynthesis drives pigmentation (Figure 1g). Concurrently, chlorophyll a and the chlorophyll a/b ratio decreased significantly at the late stage in CPZ (*p* < 0.05) and were significantly lower than those in ZQ3000, whereas chlorophyll b remained unchanged, supporting the involvement of chlorophyll breakdown in spike pigmentation (Figure 1d–f).

### 2.2. Photosynthetic Performance Alterations in Leaves and Spikes During the Coloration Process of Hulless Barley

**Figure 2:**
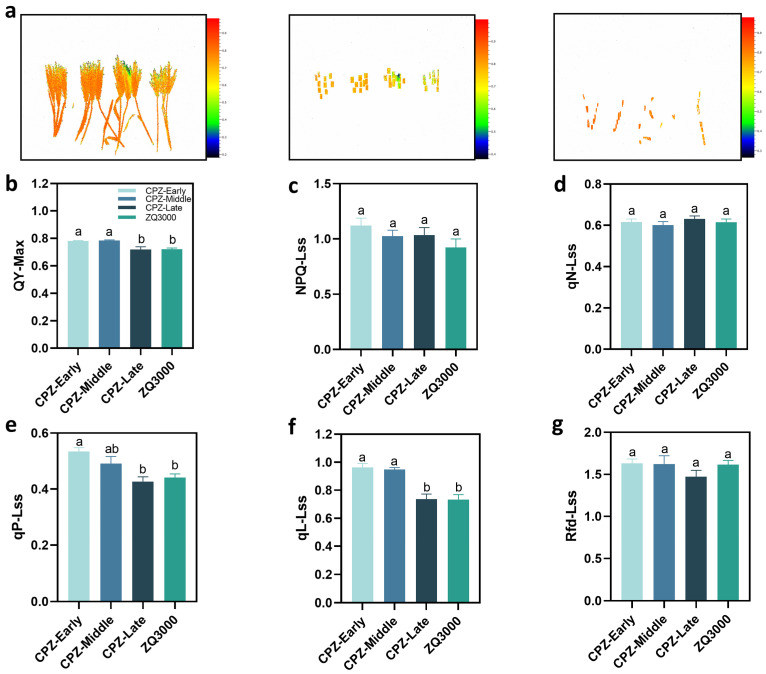
Changes in chlorophyll fluorescence parameters of spikes in hulless barley CPZ at three spike coloration stages, with non-pigmented ZQ3000 as control. (**a**) Chlorophyll fluorescence imaging of hulless barley at different coloration stages, showing QY_max_ for whole plants, spike-localized regions, and leaf-localized regions. (**b**) Maximum quantum efficiency of PSII (QY_max_); (**c**) Non-photochemical quenching (NPQ); (**d**) Non-photochemical quenching coefficient (qN); (**e**) Photochemical quenching coefficient (qP); (**f**) The fraction of PS II centers in open states (qL); (**g**) Rapid fluorescence decline parameter (Rfd). Data are shown as mean ± SE (*n* = 10). One-way ANOVA with Tukey’s test or Kruskal–Wallis with Dunn’s test was used depending on data distribution. Different lowercase letters indicate significant differences between groups at *p* < 0.05. The same letter indicates no significant difference (*p* > 0.05).

To elucidate the organ-specific photosynthetic dynamics during spike coloration in black hulless barley (CPZ), key chlorophyll fluorescence parameters associated with PSII function were measured in both spikes and leaves at the early, middle, and late spike coloration stages. The non-pigmented cultivar ZQ3000 was included as a control (Figure 2). Along with spike coloration progression, CPZ showed a gradual decline in QY_max_ in both leaves and spikes, indicating a statistically supported, modest decline in PSII photochemical efficiency (Figure 2a). The QY_max_ of CPZ spikes decreased (*p* < 0.05) during coloration, especially at late stages, while the QY_max_ of ZQ3000 was at a comparatively low level, suggesting reduced PSII performance (Figure 2b). To further evaluate the activity of the photosynthetic apparatus, qP was analyzed (Figure 2e). qP in CPZ was higher at the early stage compared to the late stage and ZQ3000 (*p* < 0.05). In line with this trend, qL, a parameter reflecting the number of open PSII reaction centers, also decreased from early to late stages (Figure 2f). These changes suggest an increasing limitation on the PSII electron acceptor side and a gradual suppression of electron transport during spike coloration.

In contrast, there were no statistically significant differences among varieties in NPQ and qN (Figure 2c,d). This suggests that energy dissipation mechanisms in spikes remained relatively stable throughout the coloration process. Similarly, the Rfd parameter showed no significant differences across the different stages or between genotypes (*p* > 0.05), indicating that this parameter remained stable during spike coloration in CPZ (Figure 2g).

**Figure 3:**
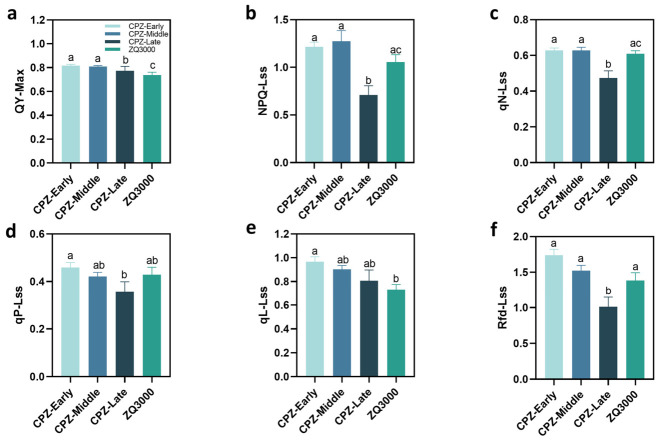
Changes in leaf chlorophyll fluorescence parameters of hulless barley CPZ at three spike coloration stages, with the non-pigmented ZQ3000 as control. (**a**) Maximum quantum efficiency of PSII (QY_max_). (**b**) Non-photochemical quenching (NPQ). (**c**) Non-photochemical quenching coefficient (qN). (**d**) Photochemical quenching coefficient (qP). (**e**) The fraction of PS II centers in open states (qL). (**f**) Rapid fluorescence decline parameter (Rfd). Data are shown as mean ± SE (*n* = 8). One-way ANOVA with Tukey’s test or Kruskal–Wallis with Dunn’s test was used depending on data distribution. Different lowercase letters indicate significant differences between groups at *p* < 0.05.

To further assess how spike coloration influences other photosynthetic organs, chlorophyll fluorescence parameters were also examined in the leaves of CPZ at the early, middle, and late spike coloration stages, using the non-pigmented white hulless barley ZQ3000 as a control (Figure 3). Significant differences in QY_max_ were observed among the three spike coloration stages in CPZ leaves and between the two accessions (Figure 3a). In CPZ, QY_max_ was significantly higher at the early and middle stages than at the late stage of spike coloration. Moreover, these values were also higher than those in ZQ3000 (*p* < 0.05). Notably, even at the late stage, CPZ exhibited higher QY_max_ than ZQ3000, indicating that despite a decline in PSII activity, CPZ retained greater photochemical efficiency than the non-coloring control. Following the changes in QY_max_, the corresponding variations in NPQ are illustrated (Figure 3b). In CPZ, NPQ at the late stage was significantly lower than that of the early and middle stages (*p* < 0.05), and also significantly lower than that in ZQ3000 (*p* < 0.05). This indicates that the energy dissipation capacity was reduced in late-stage leaves, which might be attributed to a decline in the activity of thermal dissipation mechanisms. The qN exhibited a similar trend (Figure 3c). In CPZ, both early and middle stages were significantly higher than the late stage (*p* < 0.05), while the late stage was significantly lower than ZQ3000 (*p* < 0.05), reinforcing that regulated NPQ capacity was attenuated in late-stage CPZ leaves.

In contrast, changes in qP were limited (Figure 3d), with significant differences observed between the early and late stages of CPZ (*p* < 0.05). These results indicate a slight reduction in the proportion of open PSII centers at the late stage in CPZ. Extending these observations, qL in CPZ was significantly higher than in ZQ3000 at the early stage (Figure 3e), suggesting a more oxidized QA pool and a higher fraction of open PSII reaction centers in CPZ during the early phase of spike coloration compared to ZQ3000. In addition, Rfd differed significantly among the stages in CPZ (Figure 3f). Both the early and middle stages showed significantly higher Rfd values than the late stage (*p* < 0.05), indicating a decline in photosynthetic potential during the coloration process.

Overall, chlorophyll fluorescence parameters in CPZ leaves exhibited dynamic changes during spike coloration. PSII efficiency and photosynthetic potential continuously declined, and NPQ decreased significantly at the late stage, reaching levels even lower than those in ZQ3000, indicating a marked reduction in energy dissipation capacity.

### 2.3. Differential PSII Light Responses in Leaves and Spikes During Coloration in Hulless Barley

**Figure 4:**
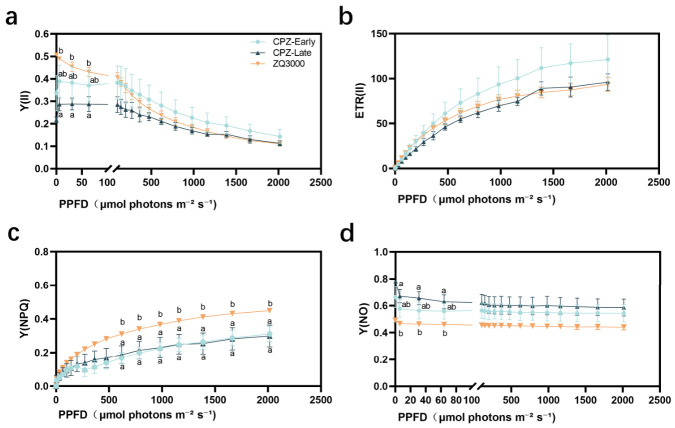
Rapid light-response curves of spike tissues in black hulless barley (CPZ) and non-pigmented white hulless barley (ZQ3000) under varying photosynthetic photon flux density (PPFD). (**a**) Effective quantum yield of PSII [Y(II)]; (**b**) Electron transport rate [ETR(II)]; (**c**) Quantum yield of regulated non-photochemical energy dissipation [Y(NPQ)]; (**d**) Quantum yield of non-regulated energy dissipation [Y(NO)]. Each panel includes 3 response curves corresponding to CPZ spikes at the early and late coloration stages and ZQ3000 spikes at the same PAR levels. Data are presented as mean ± SE (*n* = 3). Different lowercase letters at selected PPFD levels indicate significant differences among samples (*p* < 0.05, one-way ANOVA, Tukey’s test); the absence of distinct letters indicates no significant difference.

While previous measurements were conducted under constant light conditions, rapid light-response curves offer deeper insight into organ-specific responses to fluctuating light intensities. To investigate how different organs respond to variable light during the coloration process, rapid light-response measurements were first performed in the spikes of black hulless barley CPZ at the early and late coloration stages (Figure 4).

Y(II) was highest at the early coloration stage of CPZ spikes and declined to the lowest level at the late stage, indicating a reduction in PSII activity over time. The non-pigmented cultivar ZQ3000 maintained higher Y(II) at low photosynthetic photon flux density (PPFD; μmol photons m^−2^ s^−1^) but showed a sharper decrease with increasing PPFD. At PPFD = 1386 μmol photons m^−2^ s^−1^, Y(II) in ZQ3000 declined to levels comparable to those of CPZ at the late coloration stage, suggesting greater light sensitivity in ZQ3000, whereas CPZ showed a more gradual decline. Consistent with the Y(II) pattern, ETR(II) also showed a decreasing trend during the spike coloration process of CPZ, indicating a gradual decline in PSII electron transport capacity (Figure 4b). At PPFD levels below 1386 μmol photons m^−2^ s^−1^, CPZ at the early stage maintained relatively high ETR(II), while ZQ3000 showed intermediate levels, and CPZ at the late stage was the lowest. At PPFD = 1386 μmol photons m^−2^ s^−1^, ZQ3000 subsequently declined in ETR(II), dropping below CPZ at both coloration stages, suggesting a stronger down-regulation of electron transport at higher irradiance levels.

In contrast to photochemical indicators, energy dissipation mechanisms exhibited distinct patterns. Y(NPQ) varied significantly among varieties. ZQ3000 consistently showed higher Y(NPQ) than CPZ across both coloration stages, indicating a stronger capacity for regulated non-photochemical energy dissipation. In CPZ spikes, Y(NPQ) values at the early and late stages were relatively similar, suggesting limited adjustment of regulated energy dissipation during the spike coloration process (Figure 4c). To further characterize excitation-energy partitioning, we analyzed Y(NO), which represents the quantum yield of non-regulated energy dissipation. Y(NO) showed clear baseline differences among varieties (Figure 4d). In CPZ spikes, Y(NO) was higher at both the early and late stages, suggesting that a larger fraction of excitation energy was dissipated via non-regulated pathways compared with ZQ3000 across the indicated PPFD range.

**Figure 5:**
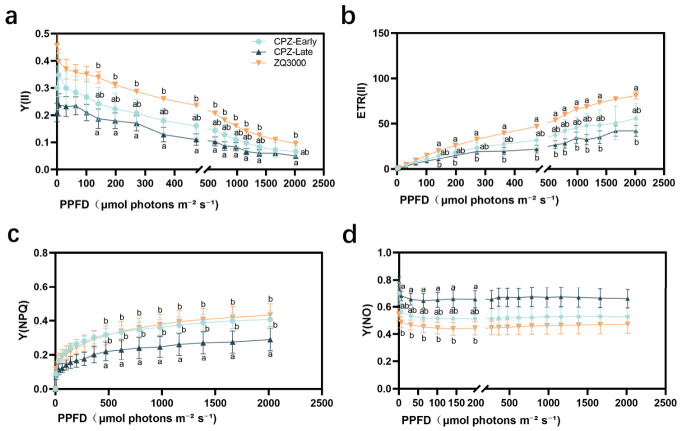
Rapid light-response curves of leaves in black hulless barley (CPZ) and non-pigmented white hulless barley (ZQ3000) under varying photosynthetic photon flux density (PPFD). (**a**) Effective quantum yield of PSII [Y(II)]; (**b**) Electron transport rate of PSII [ETR(II)]; (**c**) Quantum yield of regulated non-photochemical energy dissipation [Y(NPQ)]; (**d**) Quantum yield of non-regulated energy dissipation [Y(NO)]. Each panel includes 3 response curves corresponding to CPZ leaves at the early and late coloration stages, and ZQ3000 leaves at the same PAR levels. Data are presented as mean ± SE (*n* = 3). Different lowercase letters at selected PPFD levels indicate significant differences among samples (*p* < 0.05, one-way ANOVA, Tukey’s test); the absence of distinct letters indicates no significant difference.

Following the analysis of photosynthetic performance in spikes, we further examined the light-response characteristics in leaves. The measured parameters included Y(II), ETR(II), Y(NPQ), and Y(NO). The non-pigmented cultivar ZQ3000 served as a control (Figure 5). Across all samples, Y(II) decreased with increasing photosynthetically active radiation (PAR) (Figure 5a). At comparable light intensities, Y(II) in CPZ decreased significantly from the early to the late stage, indicating reduced PSII quantum efficiency as coloration progressed. ZQ3000 maintained consistently higher Y(II) across all light intensities compared to CPZ, indicating more efficient light utilization and enhanced PSII performance. Building on this trend, the ETR(II) curves exhibited clear species-specific and stage-specific differences (Figure 5b). CPZ exhibited generally lower ETR(II) values throughout the examined stages, with partially overlapping curves and minimal shifts, reflecting relatively stable but limited electron transport capacity. In contrast, ZQ3000 maintained higher ETR(II) under all PAR levels, suggesting greater PSII electron transport efficiency and better adaptability to light stress.

Plants are vulnerable to photodamage when exposed to light intensities that exceed their photosynthetic capacity. To protect themselves, they activate NPQ, a set of processes that dissipate excess excitation energy as heat [43,44]. Changes in Y(NPQ) were assessed under different light conditions to characterize photoprotective responses (Figure 5c). Y(NPQ) increased with increasing irradiance in all accessions, and ZQ3000 consistently exhibited significantly higher Y(NPQ) than late-stage CPZ above 500 μmol m^−2^ s^−1^, indicating a greater capacity for regulated non-photochemical energy dissipation. Unregulated energy dissipation, represented by Y(NO), remained largely stable across PAR levels but differed in baseline among varieties (Figure 5d). CPZ showed higher Y(NO) than ZQ3000, indicating a greater proportion of excitation energy dissipated via non-regulated pathways under our measurement conditions, whereas ZQ3000 maintained lower Y(NO) across the tested PPFD range.

## 3. Discussion

### 3.1. Pigment Remodeling in CPZ Spikes Reflects a Shift Away from Light Harvesting

Over the past decade, the coordinated regulation of anthocyanin accumulation and chlorophyll degradation has been extensively investigated [45,46]. The progressive shift in CPZ spikes from green to purple was accompanied by changes in pigment composition. Anthocyanin content rose significantly from early to late coloration stages (Figure 1g), while chlorophyll a and the chlorophyll a/b ratio both declined (Figure 1d,f). Chlorophyll b showed no significant change (Figure 1e). This pattern points to a functional shift in spike tissue over the coloration period: light-harvesting capacity declined while anthocyanin-based pigmentation increased. This combination of declining chlorophyll a and rising anthocyanin is consistent with what has been described in other reproductive and senescing plant tissues, where pigment transitions accompany changes in the balance between photosynthesis and stress responses [45,47]. The fact that chlorophyll b remained stable suggests the loss was not a uniform degradation of all light-harvesting pigments, but a more selective change that affected chlorophyll a and the LHCII antenna complex ratio more specifically.

Whether the accumulated anthocyanins in CPZ spikes provided a meaningful optical screening effect to reduce the photon flux reaching chloroplasts is a reasonable hypothesis. Anthocyanins accumulate in the vacuoles of epidermal and subepidermal cells, where they can act as an optical filter between incoming light and the chloroplast-bearing mesophyll. By absorbing a portion of incident photons before they reach chlorophylls, this layer can reduce excitation pressure on PSII under high irradiance [48]. This mechanism is distinct from NPQ-based thermal dissipation because it operates upstream of light absorption by chlorophylls rather than downstream of it. The two strategies are therefore not directly comparable in terms of their effects on the fluorescence parameters we measured. Different anthocyanin species differ in their absorption properties [16,17], although our measurement of anthocyanins was limited to total content using the pH-differential method, and did not distinguish specific anthocyanin compounds, the observed trends align with the general antioxidant properties attributed to anthocyanins. Future studies employing HPLC or LC-MS will be necessary to identify and quantify individual anthocyanin species, which would help confirm their specific photoprotective roles and further elucidate their involvement in organ-specific responses. The temporal coincidence between rising anthocyanin levels and reduced chlorophyll content in CPZ spikes is consistent with the classical view that anthocyanins act as internal light filters and radical-scavenging antioxidants, thereby potentially reducing PSII excitation pressure and limiting oxidative stress under high light or UV-B [49]. Our results add a functional layer to this picture by showing that organ-specific photosynthetic tuning accompanies pigment accumulation. These findings also have practical implications for food and malt quality. Enhancing or suppressing anthocyanin biosynthesis through MBW or *HvGST* alleles is expected to alter both grain color and antioxidant capacity [50].

### 3.2. PSII Performance in CPZ Spikes Declined While NPQ Stayed Broadly Stable

As anthocyanin accumulated in CPZ spikes, QY_max_, qP, and qL all decreased significantly from early to late coloration stages (Figure 2b,e,f). This pattern indicates a declining fraction of open PSII reaction centers and lower maximal photochemical efficiency in the spike over time. Such a decline is not unusual in reproductive cereal tissues as grain filling progresses. Studies in wheat glumes have reported similar reductions in PSII photochemical parameters during caryopsis development and maturation [51,52]. Notably, NPQ and qN remained broadly stable across stages in CPZ spikes (Figure 2c,d), even as QY_max_, qP, and qL declined. This means the capacity for regulated thermal dissipation did not keep pace with the decline in photochemical output. In practical terms, the spike was doing less photochemistry without a corresponding increase in its NPQ-based dissipation capacity. In the context of the energy partitioning framework Y(II) + Y(NPQ) + Y(NO) = 1 [53], declines in Y(II) and Y(NPQ) necessarily leads to an increase in Y(NO). Higher Y(NO) is consistent with a greater fraction of absorbed energy entering non-regulated dissipation pathways, although direct evidence for elevated transfer to molecular oxygen would require ROS or singlet-oxygen measurements. This is precisely the pattern observed in CPZ spike light-response data (Figure 4). Elevated Y(NO) in this context is a consequence of energy that has no other outlet after photochemistry decreases and regulated dissipation stays flat. The rapid light-response data shown in Figure 4 add further detail. At low PPFD, ZQ3000 spikes started with higher Y(II) than CPZ at either coloration stage. At high PPFD (around 1386 μmol photons m^−2^ s^−1^), ZQ3000 Y(II) fell sharply and converged toward late-stage CPZ levels. ETR(II) showed a similar pattern. CPZ spikes displayed a more gradual decline than ZQ3000 at high light. This difference could reflect a reduced effective light input to CPZ chloroplasts due to anthocyanin screening, but it could also reflect differences in developmental stage or tissue physiology between the genotypes.

ZQ3000 spikes maintained consistently higher Y(NPQ) than CPZ across both coloration stages and across the full PPFD range (Figure 4c). CPZ spikes showed correspondingly higher Y(NO) (Figure 4d). Within the Y(II) + Y(NPQ) + Y(NO) = 1 framework [53,54], Y(NO) represents energy lost through non-regulated pathways, which can include processes associated with reactive oxygen species generation [55]. Specifically, Y(NO) encompasses energy dissipation through fluorescence emission and non-radiative internal conversion, but also through the intersystem crossing process in which excited singlet chlorophyll transitions to a triplet state that can transfer energy to molecular oxygen, generating singlet oxygen and other reactive species [48]. A reduction in Y(II) that is not compensated by an increase in Y(NPQ) inevitably elevates Y(NO) and thereby increases the probability of this damaging energy transfer to oxygen. Higher Y(NO) in CPZ indicates that a larger fraction of absorbed energy bypassed regulated dissipation and entered these non-regulated pathways. ZQ3000 spikes, by contrast, channeled more of their absorbed energy into Y(NPQ)-based regulated dissipation. The fact that CPZ spikes had lower Y(NPQ) and higher Y(NO) relative to ZQ3000 is more consistent with reduced regulated thermal dissipation capacity in CPZ spikes, not enhanced protection. Whether the presence of anthocyanins compensates for this by reducing the incident photon flux in the first place is a question that fluorescence data alone cannot resolve. Direct measurements of light transmission through pigmented spike tissues and ROS accumulation under field conditions would be needed.

### 3.3. CPZ Leaves Exhibit a Late-Stage Decline in NPQ Distinct from the Pattern in Spikes

CPZ leaves maintained higher QY_max_ than ZQ3000 at all three coloration stages (Figure 3a), which indicates preserved PSII efficiency. Yet NPQ and qN in CPZ leaves dropped significantly at the late stage, falling below ZQ3000 levels (Figure 3b,c). Rfd also declined across stages in CPZ (Figure 3f), pointing to reduced photosynthetic potential as coloration progressed. The decline in NPQ and qN in CPZ leaves is the most notable result of the leaf analysis. NPQ reflects regulated dissipation of excess excitation energy primarily through xanthophyll-cycle-dependent mechanisms involving PsbS and antenna conformational changes [56]. Decline in NPQ/qN means this regulated capacity was weakening in CPZ leaves as the plant entered later coloration stages. This reflects a reduction in the plant’s active capacity to manage excess excitation energy via regulated thermal dissipation.

One possible explanation for the late-stage decline in CPZ leaf NPQ involves source-sink dynamics. As grain filling progresses, the developing grain acts as a strong carbohydrate sink. Previous studies have proposed that elevated sink demand promotes the rapid export of photoassimilates from source tissues, maintaining a high effective electron transport rate and thereby reducing the need for NPQ activation [48]. As the grain approaches maturity and sink demand changes, this balance may shift in a way that reduces the apparent NPQ capacity measured under our actinic light conditions. An alternative explanation is that the late-stage decline in CPZ leaf NPQ reflects genuine attenuation of qE capacity. For example, through reduced PsbS levels or changes in the xanthophyll cycle pool which would be associated with advancing leaf senescence. These two explanations lead to different predictions about PSII integrity, and distinguishing between them would require biochemical analysis of PsbS and xanthophyll composition alongside the fluorescence data. The decline also reflects a natural developmental senescence of the leaf’s photoprotective machinery as resources are redistributed toward grain filling [57]. However, we cannot rule out that it also reflects phenological progression in CPZ relative to ZQ3000 at the same calendar timepoint.

The leaf rapid light-response curves (Figure 5) are consistent with this reading. ZQ3000 leaves maintained higher Y(II) and ETR(II) than CPZ at all PPFD levels. ZQ3000 also showed higher Y(NPQ) (Figure 5c), while CPZ leaves had higher Y(NO) (Figure 5d). Again, higher Y(NO) in CPZ leaves indicates that more absorbed energy was dissipated through non-regulated pathways. This pattern across the full PAR range is informative because qE induction is light intensity-dependent. At low PAR, qE is minimal and Y(NPQ) differences between genotypes should be small. The fact that ZQ3000 showed higher Y(NPQ) than CPZ from moderate PAR upwards suggests that ZQ3000 leaves had a stronger capacity to induce energy-dependent thermal dissipation as excitation pressure increased. The pattern mirrors what we observed in spikes: CPZ shows lower regulated dissipation and higher non-regulated dissipation relative to ZQ3000. What these leaf results make clear is that CPZ leaves and CPZ spikes do not follow identical patterns. In spikes, NPQ and qN remained stable while QY_max_, qP, and qL declined progressively. In leaves, QY_max_ also declined significantly from early/middle to late stages within CPZ, but CPZ leaves retained higher QY_max_ than ZQ3000 throughout, whereas NPQ and qN in CPZ leaves dropped at the late stage to levels below ZQ3000. These are two distinct responses, and it would be incorrect to characterize both as reflecting the same photoprotective strategy.

The results of this study support several specific findings. In CPZ spikes, PSII photochemical efficiency (QY_max_, qP, qL) declined progressively across coloration stages, while NPQ and qN remained broadly stable. At the light-response level, CPZ spikes showed a more gradual Y(II) decline at high PPFD compared to ZQ3000, and ZQ3000 spikes consistently showed higher Y(NPQ) and lower Y(NO). In CPZ leaves, QY_max_ was higher than ZQ3000 through all three stages, but NPQ and qN fell at the late stage to levels below ZQ3000. The two organs therefore showed distinct patterns of fluorescence change during the same coloration period. For the within-CPZ comparison, stage classification was based on the visible pigmentation phenotype of individual plants sampled on the same day. This is internally consistent, and the biological gradient it captures is real. For the CPZ vs. ZQ3000 comparison, we cannot guarantee that the two genotypes were at equivalent phenological stages at any given sampling point. ZQ3000 was sampled by calendar date, not by an equivalent phenological marker, because ZQ3000 does not undergo visible spike coloration. This makes the inter-genotype comparison less straightforward than it might appear.

The interpretive scope of this study is bounded by several limitations that warrant explicit acknowledgement. The fluorescence partitioning framework places anthocyanin-mediated optical screening and NPQ-based thermal dissipation in fundamentally different positions in the energy pathway. Whether the anthocyanins in CPZ spikes provided a net photoprotective benefit relative to the NPQ-based strategy seen in ZQ3000 is worth investigating. Addressing this question requires the following considerations. First, ZQ3000 was sampled by calendar date rather than by a phenology-equivalent marker, because ZQ3000 does not undergo visible spike coloration that would allow phenotype-based stage matching to CPZ. Inter-genotype contrasts are therefore time-matched rather than stage-matched. Future studies should incorporate post-anthesis day counts or grain moisture measurements to enable stage-matched comparisons. Second, the present study compares two genotypes. Confirmation across additional pigmented landraces, including purple-grained accessions and other white landraces, will be required before the patterns reported here can be generalized as features of pigmented versus non-pigmented hulless barley. Third, anthocyanins were quantified as total content using the pH-differential method, which does not resolve individual anthocyanin species. HPLC- or LC-MS-based identification of major cyanidin, delphinidin, and peonidin derivatives, alongside antioxidant assays, is needed to distinguish species-specific contributions. Fourth, the patterns of regulated energy dissipation reported here are inferred from chlorophyll fluorescence parameters and are not supported by direct molecular or biochemical measurements. Direct evidence of reduced oxidative damage under field conditions will also be required to test whether anthocyanins confer a net photoprotective benefit, including measurements of ROS accumulation, D1 protein degradation, and PSII recovery kinetics following high-light exposure. To distinguish among possible explanations for the late-stage NPQ decline in CPZ leaves, several candidate causes need to be ruled out, including reduced PsbS abundance, changes in the size or de-epoxidation level of the xanthophyll cycle pool, and lower NPQ requirement caused by weakened carbon sink capacity. Several experimental approaches will be required to address these limitations, including immunoblot analysis of the PsbS protein, HPLC measurement of violaxanthin, antheraxanthin, and zeaxanthin contents, in situ detection of reactive oxygen species via DAB and NBT staining along with MDA quantification, and analysis of D1 protein turnover dynamics. These analyses, together with expanded biological replication and stage-matched cross-genotype sampling, are planned for the follow-up study. Despite these limitations, the organ-specific differences documented here are internally consistent and supported by replicated measurements. Spikes and leaves of CPZ responded differently across the coloration stages, and CPZ and ZQ3000 displayed distinct energy-partitioning strategies at the PSII level. These differences are informative, and the question of whether anthocyanins confer a photoprotective advantage over regulated NPQ requires further experimental support.

## 4. Materials and Methods

### 4.1. Plant Materials

In this study, two spring barley genotypes with similar maturation time were used: Cai Peng Zi (CPZ), a representative landrace of black hulless barley characterized by spike pigmentation at maturity, and Zang Qing 3000 (ZQ3000), a widely promoted white hulless barley cultivar with consistently non-pigmented spikes. These two genotypes were selected to investigate organ-specific differences in photoprotective strategies, with CPZ exhibiting spike pigmentation due to anthocyanin accumulation and ZQ3000 serving as a non-pigmented control under high light field conditions on the Qinghai–Tibet Plateau. The two genotypes were grown in adjacent field plots. Both genotypes were sown on 13 April 2023, and harvested on 30 August 2023, at the experimental station of the Institute of Agriculture, Tibet Academy of Agriculture and Animal Husbandry Sciences (Lhasa, Tibet, China; 29.6532° N, 91.1026° E; 3650 m above sea level). All plants were cultivated under identical soil fertility, tillage practices, and climatic conditions to ensure uniformity of environmental factors. During July to August 2023, the experimental site in Lhasa, Tibet, experienced an average midday PAR of approximately 1800–2000 μmol photons m^−2^ s^−1^ under clear-sky conditions. The site receives full-spectrum natural sunlight, with average daytime temperatures ranging from 12 °C to 25 °C and nighttime temperatures between 8 °C and 13 °C. At noon, under clear skies, the solar spectrum ranged from 380 to 780 nm, covering the full PAR range (400–700 nm), with all values normalized to peak intensity (Supplementary Figure S1). The region features long day-light durations (12–13 h/day), characteristic of the high-altitude Tibetan Plateau, providing a high-radiation but low-humidity environment ideal for crop growth. No fertilizer or irrigation was applied beyond natural precipitation. In July 2023, the mean temperature was 22 °C, and total precipitation reached 669.1 mm (China Meteorological Data Service Center).

The two genotypes were grown in adjacent plots without sub-plots. Each plot measured 5 m × 4 m (20 m^2^). Plants were sown in rows with a spacing of 0.25 m between rows and 0.1 m within rows, resulting in 15 rows per plot and approximately 50 plants per row (about 700 plants per plot). Border rows and plants at both ends of each row were excluded from sampling to avoid edge effects. CPZ plants grown in the same field plot exhibited visible variation in spike pigmentation due to natural phenotypic heterogeneity. On the same sampling day, CPZ individuals were visually classified into three anthocyanin accumulating stages—early, middle, and late—based on the progression of spike coloration from green to purplish-red. For each stage, three CPZ individuals were randomly selected from the central area of the plot using a random number table, ensuring that selected plants were not shaded by neighbors and had uniform growth. These three individuals represented three biological replicates per stage. ZQ3000, a non-pigmented white hulless barley variety with no visible changes in spike coloration, was sampled at the same time and served as a control. Three ZQ3000 plants were also randomly selected from the adjacent plot following the same randomization procedure. Because ZQ3000 exhibits no visible spike pigmentation throughout the growing season, developmental staging analogous to CPZ’s coloration-based classification was not applicable. ZQ3000 plants were therefore sampled synchronously by calendar date alongside CPZ individuals, serving as a contemporaneous non-pigmented reference under identical environmental and agronomic conditions. This design allows comparison of pigmented versus non-pigmented genotypes at the same timepoint. All plants were grown under uniform agronomic management and shared site conditions, ensuring sample consistency. Sampling was always performed between 9:00 and 10:00 AM to avoid diurnal variation.

### 4.2. Determination of Anthocyanin Content

The fresh spike samples were first weighed to determine and record their fresh weight. Subsequently, the spikes were rapidly ground into a fine powder in liquid nitrogen. A pre-chilled 2 mL centrifuge tube and spatula were used to transfer the powdered sample into the tube, and the sample weight was recorded accurately. An HCl solution (pH 2–3) was added at a ratio of 0.2 g/mL, followed by thorough mixing and incubation at room temperature for 5–10 min. The mixture was then centrifuged at 16,000 rpm for 6 min at room temperature.

The sample with the most intense pigmentation was selected to determine the appropriate dilution factor. It was diluted with potassium chloride (KCl) solution (pH 1.0) at ratios of 1:5 and 1:10, and absorbance was measured at 520 nm and 700 nm using a NanoDrop spectrophotometer (Thermo Scientific, Waltham, MA, USA). Based on these results, the optimal dilution factor was applied to all samples, which were then diluted with KCl solution (pH 1.0) and sodium acetate (NaAc) solution (pH 4.5), respectively. The total anthocyanin content was determined by measuring absorbance at 520 nm and 700 nm using the pH differential method [58,59].

$$A=\left(A520-A700\right)pH1.0-\left(A520-A700\right)pH4.5$$

$$C=\frac{A\times MW\times DF\times 1000}{\varepsilon \times l}$$

$$Totalanthocyanins\;(mg/g)=\frac{C\times V}{M\times 1000}$$

$$Totalanthocyanins\;(mg/g)=\frac{A\times MW\times DF\times V}{\varepsilon \times l\times M}$$

where *A* is the absorbance, *MW* is the molecular weight of cyanidin-3-glucoside (449.2 g/mol), *DF* is the dilution factor, *ε* is the molar extinction coefficient (26,900 L·mol^−1^·cm^−1^), *l* is the path length of the cuvette (1 cm), *V* is the extract volume (mL), and *M* is the fresh weight of the sample (g).

### 4.3. Determination of Chlorophyll Content

Fresh plant tissues were accurately weighed using an analytical balance, and the fresh weight (FW) was recorded. The samples were then thoroughly ground in a mortar and pestle. Following homogenization, an appropriate volume of 80% (*v*/*v*) acetone was added to the sample. After thorough mixing, the mixture was centrifuged at 10,000 rpm for 3 min. The resulting supernatant was collected and transferred into a cuvette. Absorbance values at 663 nm and 645 nm (denoted as A663 and A645, respectively) were measured using a NanoDrop spectrophotometer (Thermo Fisher Scientific, Waltham, MA, USA).

Chlorophyll concentrations were calculated using the following equations [60], and expressed as milligrams per gram of fresh weight (mg/g FW):

$$Totalchlorophyll\left(mg/gFW\right)=\left(8.02\times A663+20.21\times A645\right)\times \frac{V}{FW}$$

$$Chlorophyll\;a\;(mg/gFW)=(12.7\times A663-2.69\times A645)\times \frac{V}{FW}$$

$$Chlorophyll\;b\;(mg/gFW)=(22.9\times A645-4.68\times A663)\times \frac{V}{FW}$$

where *V* is the volume of 80% acetone used for extraction (mL), and *FW* is the fresh weight of the sample (g).

These equations correct for the overlapping absorbance of chlorophyll a and b in the visible spectrum, enabling accurate quantification of individual and total chlorophyll contents in plant tissues.

Spectrophotometric measurements for both chlorophyll and anthocyanin quantification were conducted using a NanoDrop 2000 spectrophotometer (Thermo Scientific, Waltham, MA, USA). This method allows for rapid detection with minimal sample volume requirements. However, limitations associated with NanoDrop include the short fixed optical path length (0.2 or 1 mm) and potential sensitivity to sample turbidity or particulate matter, which may affect absorbance accuracy, particularly for complex plant extracts. To minimize these effects, all samples were carefully centrifuged and visually inspected for clarity prior to measurement.

### 4.4. Chlorophyll Fluorescence Measurement and Imaging

Chlorophyll fluorescence parameters were measured following the procedure described by Nedbal et al. [61]. Prior to measurement, the plant samples were collected from the field and immediately transported to the laboratory. Samples were dark-adapted for 30 min to ensure that all PSII reaction centers were fully open. Chlorophyll fluorescence measurements were then performed using a FluorCam 800-C imaging system (PSI, Brno, Czech Republic) under controlled environmental conditions at room temperature of 25 ± 1 °C in a completely darkened laboratory to eliminate ambient light interference. The instrument was calibrated prior to each measurement to standardize the fluorescence detection area across samples.

A measuring light (<1 μmol m^−2^ s^−1^) was applied to determine the minimal fluorescence (Fo), followed by a saturating pulse of 7000 μmol m^−2^ s^−1^ lasting 800 ms to obtain maximal fluorescence values (Fm or Fm′). An actinic light of 1200 μmol m^−2^ s^−1^ was applied continuously for 5 min to induce photosynthesis prior to determining steady-state fluorescence (Fs) and light-adapted parameters. All fluorescence parameters were calculated automatically using the FluorCam software, version 1.2.4.4, under the standard “Kinetics” mode provided by the manufacturer:

QY_max_ (Fv/Fm) = (Fm − Fo)/Fm

qP = (Fm′ − Fs)/(Fm′ − Fo′)

qL = qP × (Fo′/Fs)

NPQ = (Fm − Fm′)/Fm′

qN = 1 − (Fm′ − Fo′)/(Fm − Fo)

Rfd = (Fm − Fo)/Fo

These formulas were based on standard methodologies as described by Maxwell [62]. All parameters were automatically calculated using the FluorCam software under the standard data processing mode. These fluorescence indices were used to quantitatively assess the dynamic changes in photosynthetic capacity and light energy regulation in different genotypes and organs during the grain maturation process. For each sample, multiple regions of interest (ROIs) were selected to improve data representativeness, with 10 ROIs per spike and 8 ROIs per leaf used for fluorescence signal quantification.

Based on fluorescence imaging, several key parameters related to PSII performance were quantified, including: Maximum quantum yield of PSII photochemistry (QY_max_); Photochemical quenching coefficient (qP); a parameter estimating the fraction of PSII centers in open states based on a lake model for the photosynthetic unit (qL); Non-photochemical quenching (NPQ); Non-photochemical quenching coefficient (qN); Fluorescence decrease ratio (Rfd).

### 4.5. Rapid Light Response Curve Measurement Using PAM-2500

Rapid light curves are used to study the physiological flexibility of plants’ photosynthetic units to rapid changes in irradiation, similar to what occurs in natural environments. Rapid light-response curves were measured using a PAM-2500 chlorophyll fluorometer (Walz, Effeltrich, Germany). All measurements were conducted outdoors on intact, living plants under ambient environmental conditions. All measurements were performed on intact, living plants under ambient environmental conditions. During the measurement periods (9:00–10:00 AM on clear days), the PAR at the spike level was approximately 1800–2000 μmol·m^−2^·s^−1^, and the ambient temperature was around 25 °C. No rainfall occurred during the sampling days. Prior to measurements, samples were dark-adapted for 30 min using dark-adaptation leaf clips (supplied with the PAM-2500 fluorometer; Walz, Effeltrich, Germany). Prior to initiating the light-response curve, the basal steady-state fluorescence (Ft) was monitored, and measurement was started only when Ft stabilized in the range of 0.2–0.3. This threshold was applied uniformly to both leaf and spike tissues to ensure measurement comparability. The measurements were conducted using the PAM-2500’s built-in ‘Light Curve’ protocol. Samples were sequentially exposed to a series of 17 increasing actinic light intensities (PPFD): 0, 6, 31, 64, 101, 141, 198, 271, 363, 474, 619, 785, 981, 1160, 1386, 1663, 2015 μmol photons m^−2^ s^−1^. At each light step, the instrument applied a saturating light pulse of 7000 μmol photons m^−2^ s^−1^ lasting 800 ms to determine Fm’. The following chlorophyll fluorescence parameters were recorded automatically at each step:

Effective quantum yield of PSII [Y(II)];
Electron transport rate [ETR(II)];
Quantum yield of regulated non-photochemical energy dissipation [Y(NPQ)];
Quantum yield of non-regulated energy dissipation [Y(NO)];
Y(II) + Y(NPQ) + Y(NO) = 1.

Each treatment group included three biological replicates. In each experimental run, photosynthetic responses were recorded from all three biological replicates and averaged to generate a representative light-response curve at each light intensity level.

### 4.6. Statistical Analysis

Statistical analyses were conducted using GraphPad Prism (version 8.0.1; GraphPad Software, San Diego, CA, USA). The Shapiro–Wilk and Bartlett’s tests were used to evaluate normality and variance homogeneity, respectively. For comparisons involving more than two groups, one-way ANOVA followed by Tukey’s multiple comparison test was applied when assumptions of normality and equal variance were met; otherwise, the Kruskal–Wallis test with Dunn’s multiple comparisons was used. For comparisons between two groups only, an unpaired two-tailed Student’s *t*-test was performed. A *p*-value < 0.05 was considered statistically significant.

## 5. Conclusions

Under high and variable irradiance typical of the Tibetan Plateau, hulless barley exhibits a coordinated, organ-level adjustment that couples pigment remodeling with distinct photosynthetic strategies in spikes and leaves. During the spike color transition of the black hulless barley landrace CPZ, anthocyanins accumulate while chlorophyll a and the a/b ratio decline, indicating a shift from light harvesting to altered light utilization and attenuation in reproductive tissues. Concomitantly, PSII performance in spikes decreases across stages: QY_max_, qP, and qL fall from early to late coloration, whereas NPQ and qN remain largely unchanged, pointing to reduced photochemical capacity with relatively stable energy dissipation in the spikes. Leaves, in contrast, retained higher QY_max_ than the white control ZQ3000 but showed reduced NPQ and qN at the late stage, suggesting a weaker capacity for regulated thermal dissipation in leaves at the late spike coloration stage. Taken together, the results document an organ-level pattern in which pigment changes in spikes occur alongside distinct fluorescence responses in spikes and leaves under the high and variable irradiance of the Tibetan Plateau. Anthocyanin accumulation in spikes is associated with pigment-based light attenuation and modified excitation energy partitioning, while leaves, particularly in CPZ, exhibit a reduced capacity for dynamic energy dissipation at later stages. This dual strategy may enable Qingke to maintain photosynthetic performance and grain development under extreme light stress. Our findings not only advance understanding of pigment–photosynthesis interactions but also provide potential targets for breeding anthocyanin-rich Qingke varieties with optimized light use efficiency.

## Data Availability

The original contributions presented in this study are included in the article/Supplementary Material. Further inquiries can be directed to the corresponding author.

## Supplementary Materials

The following supporting information can be downloaded at: https://www.mdpi.com/article/10.3390/plants15101489/s1, Figure S1: Normalized solar spectral distribution measured at 12:00 p.m. under clear-sky conditions in Lhasa, Tibet. The spectrum covers 380–780 nm, including the full PAR region (400–700 nm).

## Abbreviations

The following abbreviations are used in this manuscript:

PSII	Photosystem II
PPFD	Photosynthetic photon flux density (μmol photons m^−2^ s^−1^)
QY_max_	Maximum quantum efficiency of PSII (F_v_/F_m_)
qP, qL	Photochemical quenching calculated based on the puddle and lake model, respectively; the fraction of PS II centers in open states
qN	Non-photochemical quenching coefficient
Rfd	Fluorescence decrease ratio
Y(II)	Quantum yield of photosystem II photochemistry
NPQ	Non-photochemical quenching
ETR(II)	Electron transport rate through PSII
Y(NO)	Quantum yield of non-regulated energy dissipation
Y(NPQ)	Quantum yield of regulated non-photochemical quenching

## References

[1] Zeng X., Guo Y., Xu Q., Mascher M., Guo G., Li S., Mao L., Liu Q., Xia Z., Zhou J. (2018). Origin and evolution of qingke barley in Tibet. Nat. Commun..

[2] Wang J., Li H., Yang L., Li Y., Wei B., Yu J., Feng F. (2017). Distribution and translocation of selenium from soil to highland barley in the Tibetan Plateau Kashin-Beck disease area. Environ. Geochem. Health.

[3] Ramakrishna R., Sarkar D., Schwarz P., Shetty K. (2017). Phenolic linked anti-hyperglycemic bioactives of barley (*Hordeum vulgare* L.) cultivars as nutraceuticals targeting type 2 diabetes. Ind. Crops Prod..

[4] Zeng X., Yuan H., Dong X., Peng M., Jing X., Xu Q., Tang T., Wang Y., Zha S., Gao M. (2020). Genome-wide Dissection of Co-selected UV-B Responsive Pathways in the UV-B Adaptation of Qingke. Mol. Plant.

[5] Niu L., Bo L., Chen S., Qin Z., Dondup D., Namgyal L., Quzong X., Ga Z., Zhang Y., Shi Y. (2025). Comprehensive Evaluation and Construction of Drought Resistance Index System in Hulless Barley Seedlings. Int. J. Mol. Sci..

[6] Dondup D., Yang Y., Xu D., Namgyal L., Wang Z., Shen X., Dorji T., Kyi N., Drolma L., Gao L. (2023). Genome diversity and highland-adaptative variation in Tibet barley landrace population of China. Front. Plant Sci..

[7] Jiang D., Chen S., Qin Z., Bo L., Niu L., Zhou H., Wang J., Dondup D., Hou X. (2025). Deciphering drought response mechanism in Tibetan qingke through comprehensive transcriptomic and physiological analysis. Front. Plant Sci..

[8] Wu Y., Liu Y., Wang S., Zhang Y., Hou D., Ren F., Zhou S. (2025). Qingke (Highland Barley) from Qinghai-Tibet Plateau: Nutritional composition, phenolics profiles, antioxidant activity and hypoglycemic activity. J. Food Meas. Charact..

[9] Ao F., Wu J., Qiu R., Zhao H., Li L., Zong X. (2024). Preliminary research on the flavor substance and antioxidant capacity of beers produced with baking Qingke. Food Chem. X.

[10] Vasko N.I., Mykhailenko Y.O. (2024). Quality Properties of Naked Barley and Caryopsis Color Inheritance. Plant Breed. Seed Prod..

[11] Zhang Y., Sun J., Ge Z., Liang Q., Zhao H., Ye L., Zhang G., Cai S. (2024). Genetic regulation of anthocyanin biosynthesis in barley: Insights into purple pigmentation in the Zipi genotype. Plant Growth Regul..

[12] Shoeva O.Y., Mock H.P., Kukoeva T.V., Börner A., Khlestkina E.K. (2016). Regulation of the Flavonoid Biosynthesis Pathway Genes in Purple and Black Grains of *Hordeum vulgare*. PLoS ONE.

[13] Wang Y., Chen L., Yao Y., Chen L., Cui Y., An L., Li X., Bai Y., Yao X., Wu K. (2024). Investigating the regulatory role of HvANT2 in anthocyanin biosynthesis through protein-motif interaction in Qingke. PeerJ.

[14] Choo T.-M. (2002). Genetic Resources of Tibetan Barley in China. Crop Sci..

[15] Saigo T., Wang T., Watanabe M., Tohge T. (2020). Diversity of anthocyanin and proanthocyanin biosynthesis in land plants. Curr. Opin. Plant Biol..

[16] Zhang T., Ma J., Wu X., Hao Z., Dun C., Chen C. (2021). Qualitative and semi-quantitative assessment of anthocyanins in Tibetan hulless barley from different geographical locations by UPLC-QTOF-MS and their antioxidant capacities. Open Chem..

[17] Xu D., Dondup D., Dou T., Wang C., Zhang R., Fan C., Guo A., Lhundrup N., Ga Z., Liu M. (2023). HvGST plays a key role in anthocyanin accumulation in colored barley. Plant J..

[18] Chen H., Wang M., Zhang L., Ren F., Li Y., Chen Y., Liu Y., Zhang Z., Zeng Q. (2024). Anthocyanin profiles and color parameters of fourteen grapes and wines from the eastern foot of Helan Mountain in Ningxia. Food Chem. X.

[19] Ghareaghajlou N., Hallaj-Nezhadi S., Ghasempour Z. (2021). Red cabbage anthocyanins: Stability, extraction, biological activities and applications in food systems. Food Chem..

[20] Xia D., Zhou H., Wang Y., Li P., Fu P., Wu B., He Y. (2021). How rice organs are colored: The genetic basis of anthocyanin biosynthesis in rice. Crop J..

[21] Lu N., Jun J.H., Li Y., Dixon R.A. (2023). An unconventional proanthocyanidin pathway in maize. Nat. Commun..

[22] Yao X., Yao Y., An L., Li X., Bai Y., Cui Y., Wu K. (2022). Accumulation and regulation of anthocyanins in white and purple Tibetan Hulless Barley (*Hordeum vulgare* L. var. *nudum* Hook. f.) revealed by combined de novo transcriptomics and metabolomics. BMC Plant Biol..

[23] Lakshmikanthan M., Muthu S., Krishnan K., Altemimi A.B., Haider N.N., Govindan L., Selvakumari J., Alkanan Z.T., Cacciola F., Francis Y.M. (2024). A comprehensive review on anthocyanin-rich foods: Insights into extraction, medicinal potential, and sustainable applications. J. Agric. Food Res..

[24] Hasan M.M., Mia M.S., Yang J., Zeng Y., Yang T. (2025). Molecular mechanisms of how black barley accumulates higher anthocyanins than blue barley following transcriptomic evaluation and expression analysis of key genes in anthocyanins biosynthesis pathway. Front. Plant Sci..

[25] Lee C., Han D., Kim B., Baek N., Baik B.-K. (2013). Antioxidant and anti-hypertensive activity of anthocyanin-rich extracts from hulless pigmented barley cultivars. Int. J. Food Sci. Technol..

[26] Merzlyak M.N., Chivkunova O.B., Solovchenko A.E., Naqvi K.R. (2008). Light absorption by anthocyanins in juvenile, stressed, and senescing leaves. J. Exp. Bot..

[27] Solovchenko A. (2010). Optical Screening as a Photoprotective Mechanism. Photoprotection in Plants: Optical Screening-Based Mechanisms.

[28] Manetas Y. (2006). Why some leaves are anthocyanic and why most anthocyanic leaves are red?. Flora-Morphol. Distrib. Funct. Ecol. Plants.

[29] Stetsenko L.A., Pashkovsky P.P., Voloshin R.A., Kreslavski V.D., Kuznetsov V.V., Allakhverdiev S.I. (2020). Role of anthocyanin and carotenoids in the adaptation of the photosynthetic apparatus of purple- and green-leaved cultivars of sweet basil (*Ocimum basilicum*) to high-intensity light. Photosynthetica.

[30] Wang F., Ji G., Xu Z., Feng B., Zhou Q., Fan X., Wang T. (2021). Metabolomics and Transcriptomics Provide Insights into Anthocyanin Biosynthesis in the Developing Grains of Purple Wheat (*Triticum aestivum* L.). J. Agric. Food Chem..

[31] Tanaka A., Ito H. (2025). Chlorophyll Degradation and Its Physiological Function. Plant Cell Physiol..

[32] Li Y., Cao T., Guo Y., Grimm B., Li X., Duanmu D., Lin R. (2025). Regulatory and retrograde signaling networks in the chlorophyll biosynthetic pathway. J. Integr. Plant Biol..

[33] Humbeck K., Quast S., Krupinska K. (1996). Functional and molecular changes in the photosynthetic apparatus during senescence of flag leaves from field-grown barley plants. Plant Cell Environ..

[34] Krupinska K., Mulisch M., Hollmann J., Tokarz K., Zschiesche W., Kage H., Humbeck K., Bilger W. (2012). An alternative strategy of dismantling of the chloroplasts during leaf senescence observed in a high-yield variety of barley. Physiol. Plant..

[35] Domínguez F., Cejudo F.J. (2021). Chloroplast dismantling in leaf senescence. J. Exp. Bot..

[36] Sharma H., Sharma P., Kumar A., Chawla N., Dhatt A.S. (2024). Multifaceted Regulation of Anthocyanin Biosynthesis in Plants: A Comprehensive Review. J. Plant Growth Regul..

[37] Yu Z.-C., Lin W., Zheng X.-T., Chow W.S., Luo Y.-N., Cai M.-L., Peng C.-L. (2021). The relationship between anthocyanin accumulation and photoprotection in young leaves of two dominant tree species in subtropical forests in different seasons. Photosynth. Res..

[38] Zhao S., Blum J.A., Ma F., Wang Y., Borejsza-Wysocka E., Ma F., Cheng L., Li P. (2022). Anthocyanin Accumulation Provides Protection against High Light Stress While Reducing Photosynthesis in Apple Leaves. Int. J. Mol. Sci..

[39] Li X., Zhang X., Liu G., Tang Y., Zhou C., Zhang L., Lv J. (2020). The spike plays important roles in the drought tolerance as compared to the flag leaf through the phenylpropanoid pathway in wheat. Plant Physiol. Biochem..

[40] Molero G., Reynolds M.P. (2020). Spike photosynthesis measured at high throughput indicates genetic variation independent of flag leaf photosynthesis. Field Crops Res..

[41] Borrill P., Fahy B., Smith A.M., Uauy C. (2015). Wheat Grain Filling Is Limited by Grain Filling Capacity rather than the Duration of Flag Leaf Photosynthesis: A Case Study Using NAM RNAi Plants. PLoS ONE.

[42] Liang X., Liu Y., Chen J., Adams C. (2018). Late-season photosynthetic rate and senescence were associated with grain yield in winter wheat of diverse origins. J. Agron. Crop Sci..

[43] Zuo G. (2025). Non-photochemical quenching (NPQ) in photoprotection: Insights into NPQ levels required to avoid photoinactivation and photoinhibition. New Phytol..

[44] van Amerongen H., Croce R. (2025). Nonphotochemical quenching in plants: Mechanisms and mysteries. Plant Cell.

[45] Manetas Y., Buschmann C. (2011). The interplay of anthocyanin biosynthesis and chlorophyll catabolism in senescing leaves and the question of photosystem II photoprotection. Photosynthetica.

[46] Wang M., Chen L., Liang Z., He X., Liu W., Jiang B., Yan J., Sun P., Cao Z., Peng Q. (2020). Metabolome and transcriptome analyses reveal chlorophyll and anthocyanin metabolism pathway associated with cucumber fruit skin color. BMC Plant Biol..

[47] Hoch W.A., Singsaas E.L., McCown B.H. (2003). Resorption Protection. Anthocyanins Facilitate Nutrient Recovery in Autumn by Shielding Leaves from Potentially Damaging Light Levels. Plant Physiol..

[48] Gómez R.L., Lagorio M.G. (2025). Non-photochemical Quenching in Plants: A Chlorophyll a Fluorescence Perspective. Fluorescence of Living Plants: Basics, Applications and Trends.

[49] Lin S., Guo H., Gong J.D.B., Lu M., Lu M.-Y., Wang L., Zhang Q., Qin W., Wu D.-T. (2018). Phenolic profiles, β-glucan contents, and antioxidant capacities of colored Qingke (Tibetan hulless barley) cultivars. J. Cereal Sci..

[50] Shoeva O.Y., Zedgenizova V.D., Egorova A.A., Gerasimova S.V., Kukoeva T.V., Vasiliev G.V., Kovaleva O.N., Zakhrabekova S., Hansson M., Hertig C.W. (2025). Analysis of Anthocyanin-Less 2 Diversity in Barley Reveals a Specific Allele to Cause Purple-Colored Grains. J. Agric. Food Chem..

[51] Kong L., Sun M., Xie Y., Wang F., Zhao Z. (2015). Photochemical and antioxidative responses of the glume and flag leaf to seasonal senescence in wheat. Front. Plant Sci..

[52] Lu Q., Lu C. (2004). Photosynthetic pigment composition and photosystem II photochemistry of wheat ears. Plant Physiol. Biochem..

[53] Klughammer C., Schreiber U. (2008). Complementary PS II quantum yields calculated from simple fluorescence parameters measured by PAM fluorometry and the Saturation Pulse method. PAM Appl. Notes.

[54] Kramer D.M., Johnson G., Kiirats O., Edwards G.E. (2004). New Fluorescence Parameters for the Determination of QA Redox State and Excitation Energy Fluxes. Photosynth. Res..

[55] Baker N.R. (2008). Chlorophyll Fluorescence: A Probe of Photosynthesis In Vivo. Annu. Rev. Plant Biol..

[56] Ruban A.V., Wilson S. (2020). The Mechanism of Non-Photochemical Quenching in Plants: Localization and Driving Forces. Plant Cell Physiol..

[57] Lu Q., Lu C., Zhang J., Kuang T. (2002). Photosynthesis and chlorophyllafluorescence during flag leaf senescence of field-grown wheat plants. J. Plant Physiol..

[58] Jin H.M., Dang B., Zhang W.G., Zheng W.C., Yang X.J. (2022). Polyphenol and Anthocyanin Composition and Activity of Highland Barley with Different Colors. Molecules.

[59] Nakata M., Ohme-Takagi M. (2014). Quantification of Anthocyanin Content. Bio-Protocol.

[60] Banu N. (2015). Extraction and estimation of chlorophyll from medicinal plants. Int. J. Sci. Res..

[61] Nedbal L., Soukupová J., Kaftan D., Whitmarsh J., Trtílek M. (2000). Kinetic imaging of chlorophyll fluorescence using modulated light. Photosynth. Res..

[62] Maxwell K., Johnson G.N. (2000). Chlorophyll fluorescence—A practical guide. J. Exp. Bot..

