# Supplementary material for: Contrasting PSII Photochemistry and Energy Partitioning Between Spikes and Leaves During Grain Anthocyanin Accumulation in Hulless Barley on the Tibetan Plateau
Source: Plants (Basel). 2026 May 13;15(10):1489. doi: 10.3390/plants15101489 (PMC13211285; doi:10.3390/plants15101489)
Supplement: Supplementary file 1 [file plants-15-01489-s001.zip › plants-4237335-supplementary.pdf]

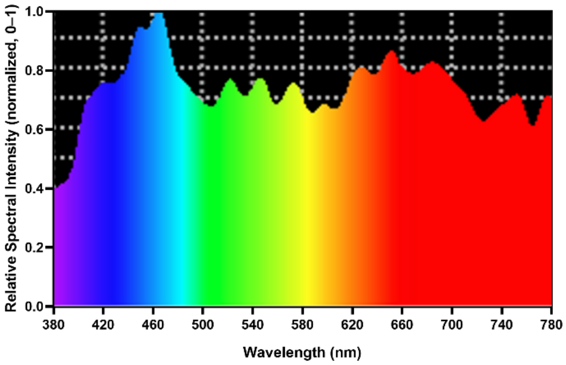

Figure S1. Normalized solar spectral distribution measured at 12:00 pm under clear-sky conditions in Lhasa, Tibet. The spectrum covers 380–780 nm, including the full PAR region (400–700 nm).
